# Genomic Comparisons in an Austral–Pacific Sandalwood (Santalaceae) Complex Highlights Novel Clades, Divergent Groups, and the Conservation Dynamics of Critically Endangered and Non‐Threatened Lineages

**DOI:** 10.1002/ece3.71246

**Published:** 2025-05-05

**Authors:** Aaron Brunton, Tony Page, David J. Lee

**Affiliations:** ^1^ Forest Research Institute University of the Sunshine Coast Maroochydore Australia; ^2^ School of Science, Technology and Engineering University of the Sunshine Coast Maroochydore Australia

**Keywords:** Australian sandalwood, conservation genomics, genomic divergence, species diversity, sustainable management, threatened species

## Abstract

Understanding the genetic processes underlying divergence and connectivity among species is crucial for identifying evolutionary histories and informing conservation strategies. The *Santalum* genus exhibits distinct genetic variations across the complex geographic regions of Australia, Asia, and the Pacific Islands. This study leveraged genome‐wide SNP markers to explore the genetic relationships within critically endangered and non‐threatened species in an Austral‐Pacific sandalwood complex, including *Santalum lanceolatum*, *S. leptocladum* , and *S. macgregorii*. Our findings revealed significant geographic partitioning and genetic divergence mostly aligned with current taxonomic classifications. However, notably, we showed *S. macgregorii* populations in Papua New Guinea (PNG) were divided into two distinct genetic groups: one in the Central and Gulf provinces and another in the Western Province, which shows a closer genetic relationship with *S. lanceolatum* from Australia's Northern Peninsula Area (NPA). This genetic connection suggests a history of secondary contact and potential hybridisation, influenced by historical land bridges and geological events. Our study highlighted that the sandalwood trees from the Western Province may represent a divergent lineage of *S. lanceolatum* ; the *S. macgregorii* populations in the Central and Gulf provinces display vicariant divergence due to geographic isolation. These insights underscore the evolutionary complexity of sandalwoods and emphasise the need for tailored conservation strategies. Our results advocate for genetic rescue programs involving NPA *S. lanceolatum* to enhance reproductive success in threatened sandalwood populations, offering crucial guidance for conservation and management efforts in Australasia.

## Graphical abstract

This study explored the genetic divergence and connectivity within the Santalum genus, focusing on endangered and non‐threatened species across Australia and Papua New Guinea. Genome‐wide SNP analysis revealed significant geographic partitioning, including two distinct S. macgregorii genetic groups in PNG, with one closely related to *S. lanceolatum* from Australia's Northern Peninsula. These findings highlight the need for tailored conservation strategies and advocate for genetic rescue programs to support threatened sandalwood populations.

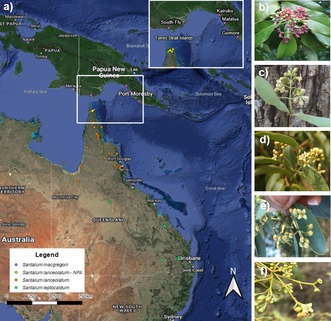

## 1 Introduction

The geological history of Australia and Southeast Asia has been shaped by dynamic shifts in sea levels and continental drift, which have played a pivotal role in the biogeographic interchange between these two regions over millions of years (Crayn et al. 2015; Chivas et al. 2001). Repeated formation of a land bridge connecting these landmasses during periods of lowered sea levels, notably during Pleistocene glacial maxima, provided corridors for the exchange of diverse flora and fauna (Steenis 1979). Conversely, during interglacial periods when sea levels rose, these connections were submerged, leading to isolation and promoting genetic divergence among populations (Joyce et al. 2021). Today, the legacy of these complex geological and climatic interactions is evident in the distribution patterns and genetic relationships of plant species across PNG and Australia, providing insights into the enduring influence of ancient land connections on current biodiversity (Joyce et al. 2021; Yap et al. 2020).

*Santalum L*. Santalaceae (sandalwood) is a genus of hemi‐parasitic plants with 16 recognised species naturally distributed throughout Asia, Australia, and the Pacific Islands that are highly valued for the fragrant oil found in the mature heartwood (Harbaugh and Baldwin 2007). An increasing global demand for sandalwood products has led to the overharvesting of natural populations of many species within the genus (Kumar et al. 2012; Lee, Burridge, Page, et al. 2019). Consequently, the depletion of wild stocks has intensified the threats facing sandalwood populations, as commercially valuable species are exploited to meet the escalating market demand for sandalwood commodities (Thomson 2020).

*Santalum macgregorii* F. Muell. is a sandalwood species native to the southern regions of Papua New Guinea (PNG) that has been subject to extensive harvesting for its aromatic heartwood. Currently, this species faces significant conservation challenges and is categorised as Critically Endangered throughout its native habitat (Rome et al. 2020; Eddowes 1998). As such, to promote the conservation of *S. macgregorii*, a number of actions have been suggested to increase the sustainable sources of the species (Bosimbi and Bewang 2005; Gunn et al. 2002) however, efforts to initiate key elements, including genetic information, into a formal breeding program remain limited (Kiapranis 2006).

Recently, *S*. *macgregorii* accessions from the Western Province of PNG were shown to have a greater phenotypic affinity with some populations of *S. lanceolatum* from Cape York Peninsula in Queensland, Australia than *S*. *macgregorii* populations in the southeastern PNG provinces (Page et al. 2020). Therefore, investigating the molecular relationships among these taxa would not only enhance the understanding of the species' genetic composition, but also inform genetic‐based conservation strategies essential for the protection of declining *S. macgregorii* populations. Moreover, given the shared geomorphic history and geographical proximity of PNG to Australia, it suggests a plausible hypothesis of genetic relatedness between certain populations of *S. macgregorii* and the Cape York Peninsula elements of *S. lanceolatum* .

In addition, genetic research with microsatellite markers indicated *Santalum lanceolatum* R. Br. from the Northern Peninsula Area of far‐north Queensland, Australia could be a distinct lineage from its cohorts outside this region (Brunton et al. 2021). This further complicates the currently described species boundaries among a closely aligned group that also contains *Santalum leptocladum* Gand., which is more broadly distributed throughout most areas of Australia (Harbaugh 2007). Phylogenetic comparisons have supported the distinction between *S. lanceolatum* and *S. leptocladum* ; however, there is a notable geographic boundary where there are also acknowledged intergrades among the two species (Harbaugh 2007; Lee, Burridge, Page, et al. 2019). Therefore, investigating such potential genetic delineation as well as any connections could offer invaluable insights into the evolutionary dynamics and conservation prospects within this economically and culturally important Austral‐Pacific complex of sandalwood.

In this study, we used a genome‐wide sequencing platform (diversity arrays technology sequencing, DArTseq) to generate single‐nucleotide polymorphism (SNP) markers for molecular analysis to determine (1) if there are distinct genetic boundaries and evolutionary lineages within provincial accessions of *S. macgregorii* and their association with the Australian sandalwood species *S. lanceolatum* and *S. leptocladum* , and (2) highlight an improved definition of the genetic boundaries within *S. lanceolatum* from the Northern Peninsula Area and the more broadly distributed representatives of *S. lanceolatum* and *S. leptocladum* to enhance conservation activities for this sandalwood lineage.

## 2 Materials and Methods

### 2.1 Study Species and Field Sampling

**FIGURE 1:**
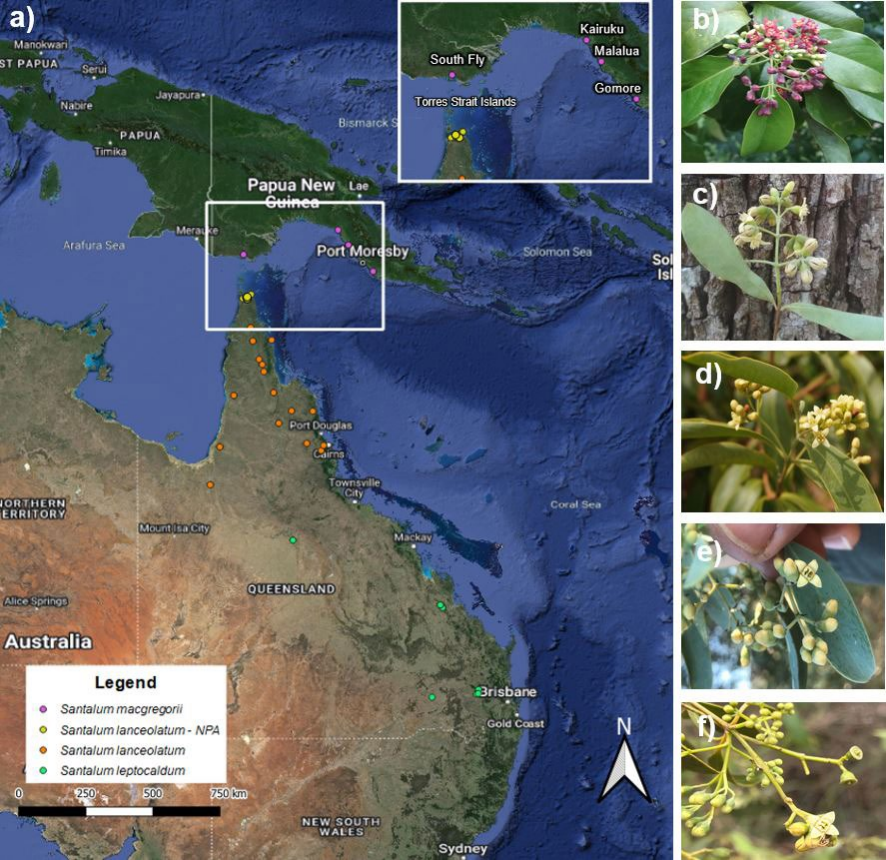
(a) Sampling distribution of an Austral‐Pacific complex of *Santalum* (sandalwood) species including; (b) *S. macgregorii* from the Gulf and Central provinces of Papua New Guinea (PNG); (c) *S. macgregorii* from the Western Province (South Fly) of PNG; (d) *S. lanceolatum* (including from the Northern Peninsula Area of Cape York Peninsula, Australia— *S. lanceolatum* —NPA) and (e) from outside the NPA region and (f) *S. leptocladum* .

*Santalum macgregorii* accessions were collected from natural populations across several provinces of Papua New Guinea (Figure 1). Provenances of *S. macgregorii* were divided into two geographically isolated groups: (1) Western Province provenances (WP) from the South Fly and (2) Gulf and Central Province provenances from Kairuku, Malalua, and Gomore. *Santalum macgregorii* from WP, Papua New Guinea, are separated by the Torres Strait, with ~150 km to Cape York Peninsula, Qld, Australia. Within the Torres Strait is a tightly clustered network of over 130 islands (Staines and Scott 2020) that could also support presently undescribed sandalwood populations (pers comm Titom Tamwoy, 2015).

In this study, 21 leaf samples of *S. macgregorii* were utilised, with nine accessions from the WP and 12 from the Gulf and Central Provinces (Table 1). Queensland accessions of *S. lanceolatum* were divided into Northern Peninsula Area (NPA), Cape York provenances and all other Queensland provenances ( *S. lanceolatum* ). A total of 59 *S. lanceolatum* germplasm resources were collected, including 18 accessions from the NPA, and 41 accessions from across the broader Cape York region of QLD. In addition, ten accessions of *S. leptocladum* were collected to assess their relationships with *S. lanceolatum* . Finally, single accessions from several closely related *Santalum* species were used as outgroups for phylogenetic analysis. For all germplasm samples, leaves were collected from trees, placed in paper bags, and then stored in airtight containers with silica gel.

**TABLE 1:** Sampling details of each accession used for genomic comparisons among an Austral‐Pacific complex of *Santalum*: *S. lanceolatum* , *S. leptocladum* and *S. macgregorii* and the outgroup representatives.

Sample id	Species	Population	*n*
BM	*S. lanceolatum*	Pipeline‐Bamaga (NPA)	3
SEI	*S. lanceolatum*	Seisia (NPA)	3
MH	*S. lanceolatum*	Muttee Heads (NPA)	2
INJ	*S. lanceolatum*	Injinoo (NPA)	3
SW	*S. lanceolatum*	Somerset West (NPA)	3
SE	*S. lanceolatum*	Somerset East (NPA)	3
RC	*S. lanceolatum*	Rocky Creek	2
DC	*S. lanceolatum*	Davies Creek	1
BM_FIS	*S. lanceolatum*	Bamaga fissured tree (NPA)	1
JAC	*S. leptocladum*	Glenmorgan	1
MMU	*S. lanceolatum*	Mt Mulligan	2
MUS	*S. lanceolatum*	Musgrave	2
LAU	*S. lanceolatum*	Laura	2
ARC	*S. lanceolatum*	Archer River	1
PAL	*S. lanceolatum*	Palmer River	2
BRA	*S. lanceolatum*	Bramwell	3
CPW	*S. lanceolatum*	Cape Weymouth	1
POR	*S. lanceolatum*	Pormpuraaw	2
HUG	*S. lanceolatum*	Hughenden	2
SUD	*S. lanceolatum*	Sudley Station	2
WOO	*S. lanceolatum*	Woondoola	2
DEL	*S. lanceolatum*	Delta Downs	2
BAT	*S. lanceolatum*	Batavia	2
CKT	*S. lanceolatum*	Cooktown	2
COE	*S. lanceolatum*	Coen	1
ST	*S. leptocladum*	Stanwell	2
KAB	*S. leptocladum*	Kabra (Moonmera)	1
MTM	*S. leptocladum*	Mt Morgan	1
COO	*S. leptocladum*	Cooyar	2
HGR	*S. leptocladum*	Highgrove	1
SCH	*S. leptocladum*	Schultz Road, Coalbank	1
SMW	*S. macgregorii*	South Fly‐ Western Province (WP)	9
SMM	*S. macgregorii*	Malalaua	3
SMK	*S. macgregorii*	Kairuku	6
SMG	*S. macgregorii*	Gomore	3
SAL	*S. album*	Cultivated collection	1
SYA	*S. yasi*	Cultivated collection	1
SAU	*S. austrocaledonicum*	Cultivated collection	1

### 2.2 SNP Genotyping and Data Filtering

Silica‐dried leaf samples were sent to for genotyping to Diversity Arrays Technology Pty Ltd. (DArT, Canberra, Australia), who performed DNA extraction and generated reduced‐representation SNP markers using the DArTseq platform. DArTseq employs a strategy similar to double‐digest RADseq, targeting thousands of SNP markers distributed across the genome (Davey et al. 2011). This approach focuses on short genomic fragments, typically 69 bp in length, which are enriched for single‐nucleotide polymorphisms representing two alleles at specific loci. By reducing genomic complexity, DArTseq enables efficient genome‐wide marker discovery. This method utilises medium sequencing density, generating approximately 1.25 million reads per sample. Genomic DNA is selectively digested with two restriction enzymes, PstI and MseI, to create a reduced representation library (Kilian et al. 2012). The libraries are sequenced on an Illumina HiSeq2000 platform, using 77 cycles to produce single‐end reads. Marker calling is performed using DArT's proprietary DS14 software, which aligns reads to each other with a tolerance of two to three nucleotide mismatches. This process identifies single‐nucleotide polymorphisms (SNPs) and presence/absence variations, referred to as SilicoDArT markers (Kilian et al. 2012). DArTseq has been extensively used in plant phylogenetic and population studies, spanning both commercial and natural populations (Steane et al. 2011; Iqbal et al. 2021; Brunton et al. 2022).

Quality filtering steps on the raw DArTseq SNP markers were implemented by an in‐house designed R package RRtools v.1.0 (Rossetto et al. 2019) to remove poor quality SNPs that have a reproducibility score of < 0.96, maximum missingness of 20%. Low‐quality samples (high proportion of missing loci) were assessed with an 80% missing loci limit to check and remove samples above this value. To reduce the possible effect of linkage disequilibrium, SNPs were filtered to retain one SNP per locus for each sample. Thus, our filtering process retained 19,851 SNPs with a mean reproducibility of 0.997. The reproducibility score for each locus was calculated by computing the mean from the locus_repro column in the filtered SNP dataset, which represents the reproducibility score for each individual locus. This score is based on the proportion of samples that exhibit data for a particular SNP at each locus. Nine *S. lanceolatum* samples and two *S. leptocladum* that presented high missingness values (> 80%) were omitted from downstream analyses. Although we applied stricter thresholds for quality control, such as increasing reproducibility to > 96%, these criteria did not lead to the exclusion of additional samples, allowing for a more balanced representation of the data while still maintaining high quality. Each filtering step was carefully designed to selectively remove poor‐quality SNPs and samples, ensuring that the final dataset accurately reflects the genomic diversity of the species studied. Applying strict missing data thresholds, for example, 0% missingness, could improve the accuracy of diversity estimates by ensuring high‐quality data (Schmidt et al. 2021) however, it can also risk excluding a significant number of samples, potentially hindering comparisons among species. To balance these considerations, we selected a threshold that retained all samples while preserving an adequate SNP count for robust analyses. This approach ensured the inclusion of a minimum of 10,000 SNPs while avoiding the substantial sample loss that a stricter threshold might have caused, thereby supporting meaningful cross‐species comparisons and maintaining analytical reliability.

To ensure that no identical genotypes were included in the SNP analysis, we performed a clonality test using the poppr package in R to run a multilocus genotype (MLG) analysis. This function allows for the identification of clonal genotypes by comparing the genetic profiles of all individuals. After applying SNP filtering, we ran the MLG analysis and retained all individuals that were represented as distinct MLGs and excluded clonal individuals within the dataset. This finding supports the conclusion that the sampled individuals were genetically unique, without the presence of clones.

Finally, we did not perform outlier loci filtering in this study. While the DArTseq platform typically captures a broad spectrum of genome‐wide variation, it may include both neutral and non‐neutral SNPs, including those potentially under selection or adaptive variants. As such, any adaptive loci present in the dataset were not specifically excluded from our analysis.

### 2.3 Phylogenetic Tree Reconstruction

Sequences of the filtered DArTseq SNPs were converted into the NEXUS format for alignment and phylogenetic analysis. We used the R packages, decipher v.2.28.0 (Wright 2016) to generate FASTA alignments of the SNP dataset, followed by phangorn v.2.11.1 (Schliep 2011) and phytools v.2.0 (Revell 2024) to construct a maximum‐likelihood tree with the best‐fit TVM + G model and nearest‐neighbour branch arrangements with 100 permutations to generate bootstrap support, representing the phylogenetic relationships for the 83 retained samples across all Santalaceae species. The best‐fit model for phylogenetic reconstruction was identified using the modelTest function to test up to 90 substitution models and identify the optimal model based on the lowest AIC and BIC values (Table S1). Optimised trees were then exported in Newick format for formatting and visualised using FigTree ver. 1.4.4 (Rambaut 2009).

SplitsTree ver. 4.41.6 (Huson 1998) was used to conduct a network analysis with a NeighbourNet tree by using the same NEXUS file that was used for the phylogenetic analysis. Default settings on uncorrected characters with ambiguous states were applied to show a visual summary of genetic relationships and to identify areas that could reflect reticulation, incomplete lineage sorting or other processes that are represented through dense networks or ‘webs’ among branches.

### 2.4 Genetic Structure and Spatial Variation

To investigate genetic structure patterns, we first explored admixture using the R package LEA v.3.12.2 (Frichot and François 2015), employing a sparse non‐negative matrix‐factorization (snmf) algorithm to derive ancestry coefficients. To ascertain the most suitable representation of *K*‐means groups, we conducted cross‐entropy analysis across *K* = 1–20 values, each with 10 runs. The resulting values of *K* were visualised in a cross‐entropy plot, with the *K* value associated with the lowest cross‐entropy prior to an increase being considered the closest estimate for the number of *K*‐means groups.

We then evaluated genetic differentiation among populations using *F*_ST_, which was calculated using the R package Hierfstat v.1.6.3 (Goudet 2005). To assess the influence of geographic distance on genetic differentiation, we used the R package vegan (Oksanen et al. 2007) to conduct a Mantle test. We used the *F*_ST_ matrix and a geographic distance based on Euclidean distance to calculate the overall effect of geographic distance on genetic distance. Finally, to further infer biogeographical scenarios of gene flow dynamics among the three sandalwood species, a relative directional migration matrix was calculated using *G*_*ST*_ (Nei and Chesser 1983) to evaluate gene flow among the regional sandalwood groups using the R shiny function, divMigrate‐online (Sundqvist et al. 2016). The statistically significant values (*α* = 0.05) were determined by bootstrapping (100 times).

## 3 Results

### 3.1 Phylogenetic Associations

**FIGURE 2:**
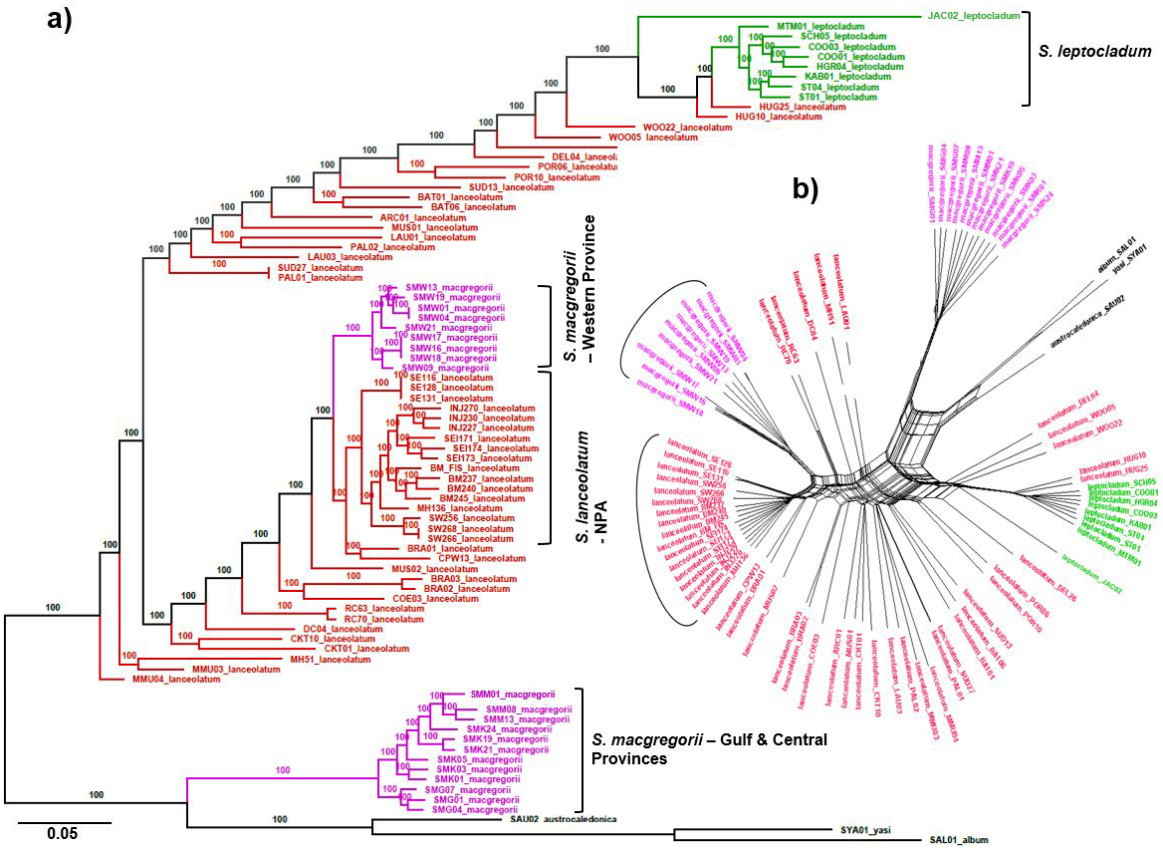
(a) Phylogenetic associations and (b) a neighbour network of an Austral‐Pacific complex of *Santalum* constructed from individually aligned and concatenated DArTseq SNP sequence markers (19,851) using a maximum‐likelihood analysis. Several congeneric sandalwood species ( *S. album* , S. *austrocaledonica* and *S. yasi*) and a more distant Santalaceae relative (*Exocarpos latifolius*) were used as outgroup representatives to root the tree and network. The values on the phylogenetic tree at the branch nodes represent bootstrap support on the basis of the best‐fit TVM + G model. *Santalum* lineages recovered are represented outside the tip labels of the tree and tips and branches are coloured by species.

The maximum‐likelihood tree we constructed showed accessions of *S. macgregorii* from the Western Province had a strong relationship (bootstrap support = 100) and formed a sister‐clade with the *S. lanceolatum* accessions from the NPA of Cape York Peninsula, Australia (Figure 2). In addition, the *S. lanceolatum* accessions from the NPA, along with the Cape Weymouth (CW‐ near Lockhart River) and Bramwell (BRA) trees, formed a distinct cluster of the phylogenetic tree. The majority of the remaining *S. lanceolatum* accessions exhibited a clustering pattern on the phylogenetic tree and network that mirrored their geographic relationships (Figure 2a,b). These patterns were also reflected in PNG accessions, which showed the *S. macgregorii* from the Central and Gulf Provinces and *S. leptocladum* positioned as distinct genetic groups. There were also two putative *S. lanceolatum* trees from Hughenden (HUG—near Flinders River) in central QLD that were positioned closely with the *S. leptocladum* group.

### 3.2 Genetic Structure and Geographic Influence

**FIGURE 3:**
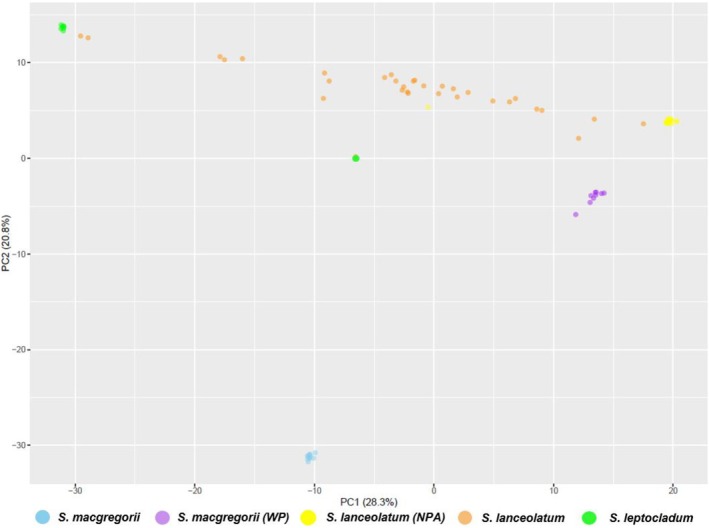
Principal component analysis (PCA) of an Austral‐Pacific clade of *Santalum* labelled based on their regional relationships; *S. macgregorii, Western Province–S. macgregorii WP, S. lanceolatum NPA* (Northern Peninsula Area), *S. lanceolatum—*from the greater Cape York Area and *S. leptocladum* generated from a set of reduced‐representation DArTseq SNP data (19,851 SNPs) showing genetic grouping along axes 1 and 2.

PCA ordination showed similar genetic relationships and clustering as the phylogenetic and network analysis that showed the *S. macgregorii* accessions from the Central and Gulf provinces as a distinct genetic group and the WP accessions closely aligned to the *S. lanceolatum* from the NPA (Figure 3). In addition, the *S. leptocladum* accessions were observed as another distinct group with several accessions (from Hughenden) of trees identified as *S. lanceolatum* closely associated (Figure 3). The *S. leptocladum* tree from Glenmorgan (JAC) was an outlier, and trees from this region may warrant further study.

These genetic structure patterns were also reflected in the comparisons of pairwise population estimates of differentiation that highlighted several distinct groups based on the regional sampling locations (Table 2.) High genetic differentiation (> 0.6) was observed between *S. leptocladum* and *S. lanceolatum* from the NPA (*F*_ST_ = 0.810) as well as the PNG accessions of *S. macgregorii* (*F*_ST_ = 0.742–0.762, Table 1). There were also moderate levels of differentiation between *S. leptocladum* and the broader *S. lanceolatum* accessions (*F*_ST_ = 0.470). There was notably limited genetic differentiation (*F*_ST_ = 0.183) between *S. lanceolatum accessions* from the NPA and *S. macgregorii* from the Western Province of PNG (Table 1). Conversely, moderate divergence (*F*_ST_ = 0.553) was present between the regional elements of *S. macgregorii* from the Western Province and those from the Gulf and Central provinces. These findings aligned with the observed phylogenetic and network associations, highlighting significant genetic divergence among these two groups.

**TABLE 2:** Pairwise *F*_ST_ matrix of an Austral‐Pacific complex of *Santalum*; *S. lanceolatum* , *S. leptocladum* and *S. macgregorii* from reduced‐representation SNP data (19,851) grouped into regional elements.

	S. leptocladum	S. lanceolatum	*S. lanceolatum* (NPA)	*S. macgregorii*	*S. macgregorii* (WP)
*S. leptocladum*	0	0.470	0.810	0.762	0.742
*S. lanceolatum*	0.470	0	0.295	0.532	0.245
*S. lanceolatum* (NPA)	0.810	0.295	0	0.732	0.183
*S. macgregorii*	0.762	0.532	0.732	0	0.553
*S. macgregorii* (WP)	0.742	0.245	0.183	0.553	0

**FIGURE 4:**
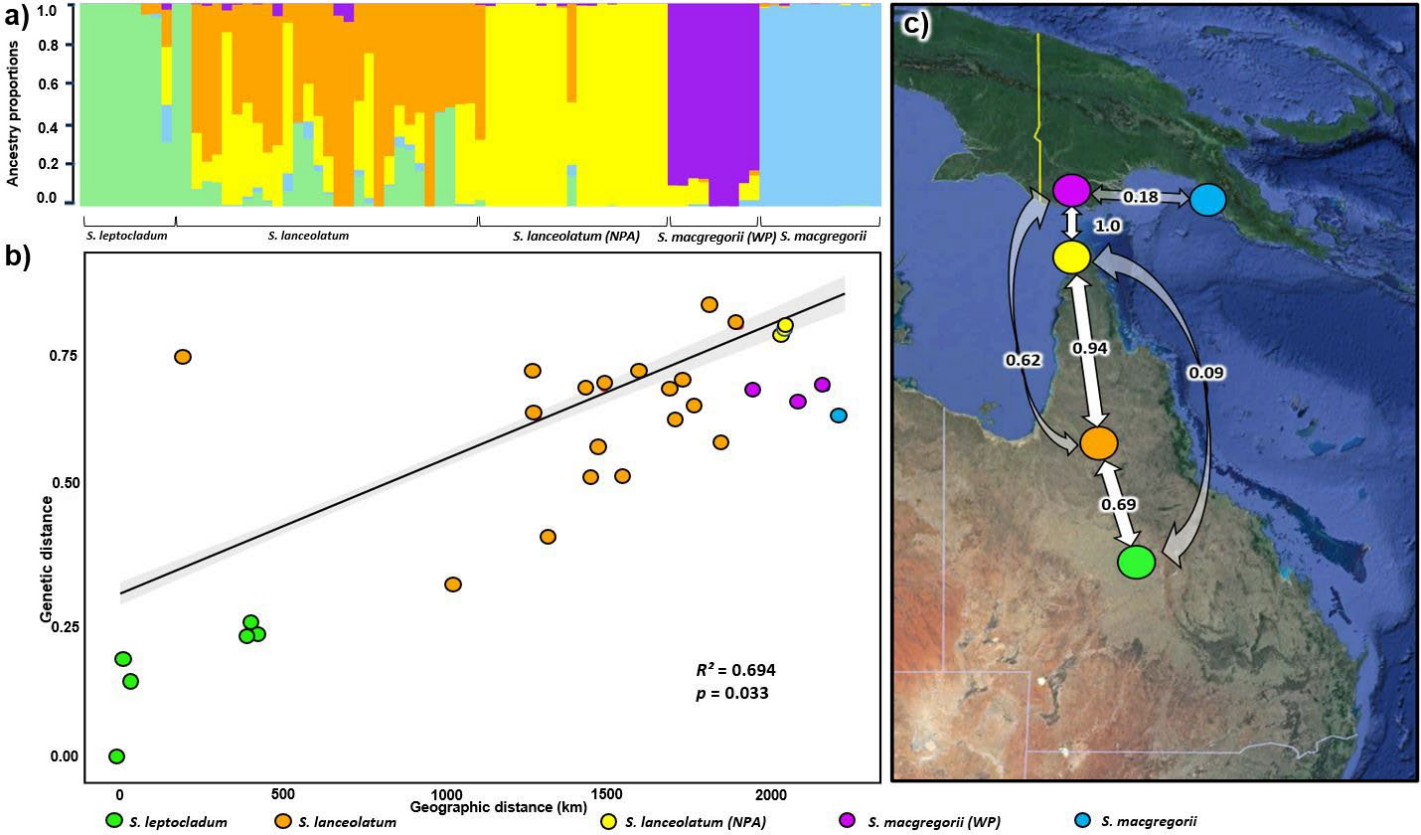
(a) Genetic admixture among an Austral‐Pacific complex of sandalwood species generated using 19,851 SNPs, showing individual assignments to ancestry proportions of five genetic clusters (*K* = 5); (b) relationship between genetic and geographic distribution (isolation by distance, IBD); and (c) significant (*α* = 0.5) directional migration rates calculated with DivMigrate online and based on *G*_*ST*_ from the three species *S. leptocladum* , *S. lanceolatum* and *S. macgregorii* coloured by region (see legends).

Admixture analysis between the study species attributed the best value of *K* to five genetic clusters (Figure S1). The assignment of individuals to each of the five clusters mostly showed discrete genetic breaks within the three species to indicate geographic partitioning; however, there was a general pattern of panmixia among the broader *S. lanceolatum* accessions (Figure 4a). Interestingly, there was one individual from the NPA sandalwood (MH) that showed genetic structure similar to those in the broader *S. lanceolatum* cluster (Figure 4a). There were also some moderate levels of admixture observed in the *S. macgregorii* WP with signals of introgression from *S. lanceolatum* from the NPA (Figure 4a). *Santalum macgregorii* individuals from eastern PNG all clustered in a distinct genetic group with negligible levels of admixture (Figure 4a).

Following the observation of genetic structure within the five clusters, the *q* values of admixture analysis were used to assess the degree of genetic contribution from each of the identified clusters to the study individuals. These values (*Q*‐scores) reflect the proportion of genetic ancestry each individual carries from the various clusters. Both regional groups of *S. macgregorii* generally showed a high degree of genetic differentiation, with most individuals predominantly belonging to a single genetic cluster. For instance, the S. *macgregorii* accessions *from* eastern PNG (Gomore, Malalua, Kairuku) displayed near‐uniform ancestry with values approaching 1.0 for a single cluster. Similarly, the Western Province individuals of S. *macgregorii* also exhibited a high degree of genetic consistency, though six accessions showed evidence of minor introgression (0.10–0.18). *Santalum lanceolatum* exhibited the highest levels of admixture among the sandalwood groups we assessed, with individuals showing a wide range of genetic proportions from different clusters, and an average admixture value of 0.30. Notably, the *S. lanceolatum* individuals from the NPA exhibited predominantly higher proportions from a single cluster, with most values ranging from 0.01 to 0.02; however, one individual (MH51) showed a high level (0.51) of admixture (Data S1). *Santalum leptocladum* also showed a high degree of differentiation, with just one individual (JAC02) observed to have a high level (0.68) of admixture.

This relationship was similar to the patterns in our isolation by distance analysis (Figure 4b). Overall, there was also strong evidence (*R* ^2^ = 0.694, *p* = < 0.05) of geographic partitioning among the species with isolation by distance (IBD) observed to have significant overall influence among the three *Santalum* species (Figure 4b) which was expected due to the large distance between *S. macgregorii* and *S. leptocladum* from mainland Australia. The relative directional migration matrix revealed several significant gene flow patterns among the sandalwood groups (Table S2). Notably, there was significant migration from the NPA *S. lanceolatum* to both the broader *S. lanceolatum* group (0.94—Figure 4c) and *S. macgregorii* from the WP (1—Figure 4c). *Santalum macgregorii* in eastern PNG was one of the most isolated, with only a limited value (0.18) of geneflow detected towards the WP sandalwood (Figure 4c). *Santalum lanceolatum* from the NPA was also highly isolated from *S. leptocladum* (0.09—Figure 4c). Interestingly, the matrix also indicated considerable migration from the WP *S. macgregorii* to the broader *S. lanceolatum* group (0.62—Figure 4c).

## 4 Discussion

Our research underscores how the dynamic geological and climatic history of the Australasian region has profoundly shaped the genetic connectivity and divergence patterns of sandalwood species. Recent evidence of phenotypic affinities among sandalwood groups from Papua New Guinea and Cape York Peninsula, Australia (Page et al. 2020), emphasises the importance of understanding genetic relationships among these species. This research is also crucial for gaining insights into the evolutionary history, intricate relationships, and guiding conservation priorities among the flora of Australia and PNG. Genomic comparisons within the Austral‐Pacific complex of *S. lanceolatum* , *S. leptocladum* , and *S. macgregorii* revealed geographic partitioning among the three species, aligning with their current taxonomic classifications. However, our genetic evidence also supports the observed phenotypic differences within *S. macgregorii*, revealing two distinct genetic groups. Populations in PNG's Central and Gulf provinces, which exhibit red flowers (seen in Figure 1b) typical of *S. macgregorii* (Page et al. 2020) and were genetically distinct from those in the Western Province, which have white flowers (seen in Figure 1c). Furthermore, the Western Province sandalwood was genetically aligned with *S. lanceolatum* from the Northern Peninsula Area (NPA) of Australia, supporting the phenotypic resemblance between these two groups. Our findings indicate secondary contact between these populations, reinforcing the phenotypic and genetic convergence.

In examining the evolutionary histories of these sandalwood species across Australasia, distinct patterns of divergence and introgression emerge, highlighting the complex dynamics of their genetic relationships. *Santalum macgregorii* in PNG's Central and Gulf provinces, along with *S. leptocladum* from Australia, showed clear evidence of vicariant divergence, supported by structural and phylogenetic analyses. This divergence indicated significant genetic differentiation was likely driven by geographic isolation over evolutionary timescales that was highlighted in the IBD and genetic structure. Conversely, *S. macgregorii* in PNG's Western Province exhibits a contrasting pattern characterised by introgression from *S. lanceolatum* in the NPA region of Australia. The geological history of the region, particularly the movements and interactions of the Sunda and Sahul plates, along with historical land bridges across the Torres Strait, has likely significantly influenced the genetic relationships and migration pathways of these species. The high bi‐directional migration rate among the NPA and Western Province sandalwood populations further supports this theory, highlighting how the geological dynamics have contributed to the observed genetic and unique distribution patterns among sandalwood species in this region.

Genetic analyses also revealed a close phylogenetic and structural relationship between Western Province sandalwood and NPA *S. lanceolatum* . Directional geneflow patterns also highlighted this association among the two regionally discrete groups, suggesting a history of secondary contact and potential hybridisation between these species. We also observed moderate levels of admixture in *S. macgregorii* individuals from Western Province, with signals of introgression from *S. lanceolatum* from the NPA. Specifically, six accessions from Western Province exhibited admixture proportions ranging from 0.10 to 0.18, indicating some level of gene flow between these populations. In contrast, individuals from eastern PNG populations of *S. macgregorii* showed negligible levels of admixture, clustering distinctly in their own genetic group. This observation prompts further questions about the timescales of secondary contact and potential hybridisation between Western Province sandalwood and *S. lanceolatum* from the NPA. Given the moderate levels of admixture observed in the Western Province individuals, it is likely that this introgression reflects more recent hybridisation events. However, the *q*‐values also suggest that this genetic exchange is not extensive, implying that hybridisation may have occurred within the last few generations, but not to the point of significant genetic homogenisation. The presence of a distinct genetic structure in the Western Province individuals also suggests that while gene flow has occurred, the populations retain distinct genetic identities.

These more recent hybridisation events are likely influenced by the shared evolutionary history of *S. macgregorii* and *S. lanceolatum* , as evidenced by the phylogenetic sister‐grouping of Western Province sandalwood with *S. lanceolatum* from the NPA. This sister‐grouping of Western Province sandalwood with *S. lanceolatum* further supports their phenotypic affinity (Page et al. 2020), indicative of a shared evolutionary history shaped by historical biogeographic events and/or contemporary dispersal events. For instance, during glacial periods of the Pleistocene/late Pleistocene (~2.5 million to 11,700 years ago) when lower sea levels formed land bridges (Chivas et al. 2001), sandalwood populations from Papua New Guinea's Western Province and *S. lanceolatum* from Australia likely facilitated gene flow and introgression between these populations. Subsequent fluctuations in sea levels and climatic shifts then isolated these populations, promoting genetic divergence. This genetic exchange may explain the strong alignment of Western Province *S. macgregorii* with *S. lanceolatum* . Based on these findings, our data strongly suggest that *S. macgregorii* from the Western Province should likely be classified as *S. lanceolatum* . The proximity of numerous islands within the Torres Strait, coupled with anecdotal reports of *S. lanceolatum* occurrences on some of these islands, suggests possible contemporary exchanges through post‐glacial cycles or cultural/trading practices involving sandalwood. However, caution is warranted, as further sampling across the Torres Strait Islands and adjacent regions is necessary to confirm this scenario and explore the potential for genetic variation or cryptic diversity within this region. These additional efforts could clarify the extent of historical and contemporary genetic exchange and refine the taxonomic relationships between the NPA sandalwood from Australia and *S. macgregorii* from the Western Province of PNG.

Notably, sandalwood populations in the NPA represent a distinct lineage separate from all other *S. lanceolatum* representatives in the study. This distinctiveness was confirmed by phylogenetic and network analyses, consistently placing NPA sandalwood as a unique evolutionary group within the broader context of *S. lanceolatum* diversity. Additionally, some sandalwood trees from the greater Cape York Peninsula (e.g., CW—Cape Weymouth) were phylogenetically associated with NPA sandalwood, reflecting the high oil properties (Page et al. 2007) shared between both provenances. This relationship was further strengthened by admixture and structure analyses. Furthermore, the presence of some sandalwood trees (Hughenden representatives) outside the typical geographic boundaries of *S. lanceolatum* (Harbaugh 2007), yet genetically aligned with *S. leptocladum* , underscores the complexity of sandalwood evolution and the potential for hybridisation across species boundaries.

Our study has provided further insights into the evolutionary processes shaping sandalwood diversity in Australasia, particularly in light of historical biogeographic events such as sea‐level fluctuations during the Pleistocene, which may have similarly influenced other taxa across the Cape York Peninsula and PNG. The genetic patterns observed in *S. macgregorii* populations from Western Province, which share a close affinity with NPA sandalwood, can be seen in a broader context of population divergence, and introgression is a common occurrence across taxa in the region, including a number of northern Australia bird species (Peñalba et al. 2019; Burley et al. 2023). For example, the introgression patterns observed in the blue‐faced honeyeater support a hypothesis of reticulate evolution across the region, with populations separated by dynamic barriers, including the now submerged land bridges and shifting coastlines between Australia and New Guinea. These biogeographic processes likely shaped the evolutionary histories of species like sandalwood, driving diversification and genetic exchange in ways that reflect past climate and sea‐level changes (Burley et al. 2023). Additionally, bird‐mediated dispersal, particularly by frugivores, may have played a key role in long‐distance seed movement across these regions. Harbaugh and Baldwin (2007) suggested that *S. lanceolatum* , originating in Australia, may have been dispersed to PNG by birds, facilitating gene flow between distant populations. This dispersal mechanism, combined with past climate events and human‐mediated processes, could explain the genetic similarity between *S. macgregorii* populations from Western Province and NPA sandalwood. Thus, the genetic affinity between these populations reflects shared evolutionary histories shaped not only by historical dispersal but also by contemporary dynamics within the region.

In terms of reproduction, *Santalum* are a dioecious genus that rely on both sexual and clonal reproduction (Tamla et al. 2011; Warburton et al. 2000) with pollination primarily achieved through insect‐mediated mechanisms (Veerendra and Padmanabha 1996). This reproductive flexibility, combined with occasional self‐incompatibility, facilitates cross‐breeding between closely related populations, allowing for hybridisation in certain circumstances (Harbaugh 2007; Subasinghe 2013). Therefore, gene flow between populations of *S. macgregorii* and *S. lanceolatum* is not only plausible but likely, especially given the observed genetic patterns and ecological conditions that support interbreeding. These dynamics of gene flow and hybridisation are consistent with the broader evolutionary processes shaping sandalwood populations, which reflect both historical and contemporary factors.

This understanding of evolutionary processes is crucial for guiding conservation efforts amidst challenges such as overharvesting, habitat loss and inadequate fire management practices for sandalwood in PNG (Gunn et al. 2002; Rome et al. 2020) and the NPA of Australia (Brunton et al. 2021; Lee, Burridge, Page, et al. 2019). *Santalum macgregorii* populations from Western Province show a close genetic affinity with NPA sandalwood, forming a distinct but genetically proximate sister‐clade. This genetic similarity reflects shared evolutionary histories shaped by historical biogeographic events and contemporary dispersal dynamics. Further exploration, including potential sampling from Torres Strait island groups, could strengthen these genetic relationships. In addition, we suggest that a taxonomic revision is necessary for a clear understanding of the phenotypic affinities and distinctions among the WP and eastern *S. macgregorii* populations and the NPA *S. lanceolatum* . This information is vital to determine the relationships among these sandalwood groups. Much like a range of sandalwood species (Butaud et al. 2005; Doran et al. 2005; Wharton 2009; Rashkow 2014), Western Province sandalwood faces numerous issues for ongoing survival in the wild, including overharvesting, reproductive failure, and limited recruitment, necessitating conservation strategies informed by genetic insights (Page et al. 2020). This is also similar to the challenges faced by the *S. lanceolatum* in the NPA region, where low seed set (Lee, Burridge, Brunton, et al. 2019) and high levels of clonality (Brunton et al. 2021) were observed. These populations also exhibit distinct traits in oil composition, leaf shape, and flower colour compared to the eastern PNG sandalwood (Page et al. 2020; Rome et al. 2020). Page et al. (2020) suggested that Western Province sandalwood may form part of a cryptic species with the *S. lanceolatum* from the NPA, approximately 150 km away from PNG. Our genetic results support this notion to underscore the importance of a genetic rescue program. As such, we advocate for the use of *S. lanceolatum* from the NPA to enhance reproductive success and conserve natural stands of WP *S. macgregorii* suffering from poor recruitment.

Historically a valuable economic resource, reviving or commencing trade of sandalwood in the WP of PNG again requires immediate conservation attention guided by genetic information. Our genetic results suggest that mixing *S. macgregorii* from the WP with *S. lanceolatum* from the NPA is likely more effective for stimulating reproductive success due to their closer genetic structure. Bringing together diverse genetic material of the NPA sandalwood has resulted in abundant seed set compared to that found in the wild (Lee, Burridge, Page, et al. 2019). While we support such a genetic rescue program, we acknowledge the need for caution due to the genetic divergence between these lineages. Our findings suggest that *S. lanceolatum* from the NPA shares enough genetic similarity with WP *S. macgregorii* to support successful gene flow and improve reproductive success. However, a controlled, gradual approach should be taken to avoid disrupting the local adaptations of WP populations. Ongoing monitoring will be crucial to prevent homogenisation and ensure the long‐term survival of locally adapted populations. Based on our results, we suggest the genetically distinct sandalwood populations from the Central and Gulf provinces of PNG are less suitable for such interventions because of their significant genetic divergence and isolation. This study also further supports the idea that the NPA sandalwood population represents a distinct lineage and should be managed as a geographically distinct race of *S. lanceolatum* . Similarly, *S. macgregorii* from eastern PNG also represents a discrete genetic lineage and requires separate conservation strategies to preserve its unique genetic identity. However, it is important to note that the SNPs used in this study were generated using the DArTseq platform, which broadly captures genome‐wide variation but may include loci under selection or non‐nuclear loci. No specific analyses were conducted to identify or exclude these loci, and this limitation should be considered when interpreting genetic patterns, particularly in contexts where adaptive variation may play a role. The inclusion of such loci could influence the interpretation of genetic diversity and population structure. Future studies could incorporate additional steps, such as mapping SNPs to reference genomes or tests for neutrality, to further refine the dataset and address these considerations. In addition, although recent studies indicate that reliable genetic estimates can often be obtained with small sample sizes when a large number of SNPs are analysed (Nazareno et al. 2017), this assumption must be applied cautiously, as species‐specific biological traits and evolutionary histories may introduce biases. In this study, the limited number of individuals sampled per species and locality in some cases may influence the resolution of population‐level patterns. Nevertheless, our sampling strategy was designed to maximise representation across the geographic range of the taxa studied, and the results should be interpreted with this limitation in mind.

## 5 Conclusion

The geomorphic relationship between Sunda and Sahul plates, along with historical and contemporary connections via land bridges such as the Torres Strait Islands, has played a pivotal role in shaping the genetic divergence and distribution patterns observed among sandalwood species from Australia and Papua New Guinea. Our study suggests that genetic mixing, particularly between taxa with poor recruitment and other genetically associated groups, is essential for the conservation and sustainability of these sandalwood species. This approach could stimulate reproductive success and help conserve natural stands, thereby enhancing the resilience and long‐term survival of these populations. Notably, the sandalwood populations in the Northern Peninsula Area (NPA) of Australia and *S. macgregorii* from the Western Province of PNG represent a distinct lineage from the broader *S. lanceolatum* group. This distinctiveness highlights the importance of managing NPA sandalwood as a geographically and genetically unique race of *S. lanceolatum* , which has implications for conservation strategies and genetic rescue programs. Similarly, sandalwood populations from eastern Papua New Guinea (PNG) form a genetically discrete group that requires separate conservation approaches. The significant genetic differentiation observed in these populations underscores the need for targeted conservation strategies to preserve their unique genetic identities, especially considering the critically endangered status of *S. macgregorii*. For instance, genetic rescue programs that incorporate *S. lanceolatum* from the NPA into the gene pool of *S. macgregorii* from the Western Province of PNG could enhance reproductive success due to their closer genetic structure. Conversely, populations from the Central and Gulf provinces of PNG, which exhibit significant genetic divergence, should be managed separately to preserve their unique genetic identities. This genetic management strategy is crucial for addressing challenges such as overharvesting and habitat loss, ensuring the long‐term conservation of sandalwood in PNG and the NPA of Australia. The implications of these findings highlight the need for a nuanced approach to the conservation of these sandalwood species, taking into account their genetic distinctiveness and the specific ecological and evolutionary pressures they face. Ensuring the survival of these distinct genetic lineages is not only vital for maintaining biodiversity, but also for sustaining the ecological and cultural heritage of the regions they inhabit.

## Author Contributions

**Aaron Brunton:** conceptualisation (equal), data curation (equal), formal analysis (lead), investigation (equal), methodology (equal), project administration (supporting), software (equal), writing – original draft (equal), writing – review and editing (equal). **Tony Page:** conceptualisation (lead), formal analysis (supporting), methodology (equal), writing – original draft (equal), writing – review and editing (equal). **David J. Lee:** conceptualisation (lead), formal analysis (supporting), investigation (equal), methodology (equal), writing – original draft (equal), writing – review and editing (equal).

## Conflicts of Interest

The authors declare no conflicts of interest.

## Supporting information


Data S1.

## Data Availability

Raw genetic data can be accessed at https://github.com/AaronBrunton/Santalum.
