# Supplementary material for: Genomic Comparisons in an Austral–Pacific Sandalwood (Santalaceae) Complex Highlights Novel Clades, Divergent Groups, and the Conservation Dynamics of Critically Endangered and Non‐Threatened Lineages
Source: Ecol Evol. 2025 May 5;15(5):e71246. doi: 10.1002/ece3.71246 (PMC12052471; doi:10.1002/ece3.71246)
Supplement: Supplementary file 1 — Data S1. [file ECE3-15-e71246-s001.docx]

Supplementray data **–** Genomic comparisons in an Austral-Pacific sandalwood (Santalaceae) complex highlights novel clades, divergent groups, and the conservation dynamics of critically endangered and non-threatened lineages

**Table S1.** Results of nucleotide substitution models used to select best-fit model of phylogenetic tree construction for three sandalwood species, *S. lanceolatum, S. leptocladum* and *S. macgregorii* from concatenation of 19,851 DArTseq markers.

| **Model** | **df** | **logLik** | **AIC** | **AICc** | **BIC** |
| --- | --- | --- | --- | --- | --- |
| JC | 163 | -310074 | 620474.7 | 620477.4 | 621760.4 |
| JC+I | 164 | -308882 | 618091.8 | 618094.6 | 619385.4 |
| JC+G(4) | 164 | -303593 | 607514.5 | 607517.3 | 608808 |
| JC+G(4)+I | 165 | -303594 | 607517.1 | 607519.9 | 608818.5 |
| F81 | 166 | -310067 | 620465.8 | 620468.7 | 621775.2 |
| F81+I | 167 | -308873 | 618080.5 | 618083.4 | 619397.8 |
| F81+G(4) | 167 | -303581 | 607496.1 | 607499 | 608813.3 |
| F81+G(4)+I | 168 | -303581 | 607498.7 | 607501.6 | 608823.8 |
| K80 | 164 | -296811 | 593950.6 | 593953.4 | 595244.2 |
| K80+I | 165 | -295499 | 591328.3 | 591331.2 | 592629.8 |
| K80+G(4) | 165 | -289657 | 579643.3 | 579646.2 | 580944.8 |
| K80+G(4)+I | 166 | -289657 | 579645.9 | 579648.7 | 580955.2 |
| HKY | 167 | -296803 | 593939.3 | 593942.1 | 595256.5 |
| HKY+I | 168 | -295486 | 591307.3 | 591310.2 | 592632.4 |
| HKY+G(4) | 168 | -289639 | 579613.2 | 579616.1 | 580938.3 |
| HKY+G(4)+I | 169 | -289639 | 579615.7 | 579618.7 | 580948.7 |
| TrNe | 165 | -296806 | 593942.9 | 593945.7 | 595244.3 |
| TrNe+I | 166 | -295493 | 591317.8 | 591320.6 | 592627.1 |
| TrNe+G(4) | 166 | -289654 | 579640.2 | 579643.1 | 580949.5 |
| TrNe+G(4)+I | 167 | -289654 | 579642.8 | 579645.7 | 580960 |
| TrN | 168 | -296801 | 593938.7 | 593941.6 | 595263.8 |
| TrN+I | 169 | -295484 | 591305.2 | 591308.1 | 592638.2 |
| TrN+G(4) | 169 | -289638 | 579614.7 | 579617.7 | 580947.7 |
| TrN+G(4)+I | 170 | -289639 | 579617.3 | 579620.2 | 580958.1 |
| TPM1 | 165 | -296810 | 593949.1 | 593951.9 | 595250.5 |
| TPM1+I | 166 | -295497 | 591326.8 | 591329.7 | 592636.1 |
| TPM1+G(4) | 166 | -289655 | 579642.2 | 579645 | 580951.5 |
| TPM1+G(4)+I | 167 | -289655 | 579644.7 | 579647.6 | 580961.9 |
| K81 | 165 | -296810 | 593949.1 | 593951.9 | 595250.5 |
| K81+I | 166 | -295497 | 591326.8 | 591329.7 | 592636.1 |
| K81+G(4) | 166 | -289655 | 579642.2 | 579645 | 580951.5 |
| K81+G(4)+I | 167 | -289655 | 579644.7 | 579647.6 | 580961.9 |
| TPM1u | 168 | -296801 | 593937.6 | 593940.5 | 595262.7 |
| TPM1u+I | 169 | -295484 | 591305.8 | 591308.7 | 592638.7 |
| TPM1u+G(4) | 169 | -289637 | 579612.1 | 579615.1 | 580945.1 |
| TPM1u+G(4)+I | 170 | -289637 | 579614.7 | 579617.7 | 580955.6 |
| TPM2 | 165 | -296788 | 593905.3 | 593908.1 | 595206.8 |
| TPM2+I | 166 | -295481 | 591293.2 | 591296 | 592602.5 |
| TPM2+G(4) | 166 | -289639 | 579610.2 | 579613 | 580919.5 |
| TPM2+G(4)+I | 167 | -289639 | 579612.7 | 579615.6 | 580929.9 |
| TPM2u | 168 | -296783 | 593902.1 | 593905 | 595227.2 |
| TPM2u+I | 169 | -295474 | 591286.2 | 591289.1 | 592619.1 |
| TPM2u+G(4) | 169 | -289632 | 579601.4 | 579604.4 | 580934.4 |
| TPM2u+G(4)+I | 170 | -289632 | 579604 | 579607 | 580944.9 |
| TPM3 | 165 | -296809 | 593947.5 | 593950.3 | 595248.9 |
| TPM3+I | 166 | -295498 | 591327.8 | 591330.7 | 592637.2 |
| TPM3+G(4) | 166 | -289656 | 579644.5 | 579647.3 | 580953.8 |
| TPM3+G(4)+I | 167 | -289657 | 579647 | 579649.9 | 580964.2 |
| TPM3u | 168 | -296799 | 593933.2 | 593936.1 | 595258.3 |
| TPM3u+I | 169 | -295484 | 591306.3 | 591309.2 | 592639.3 |
| TPM3u+G(4) | 169 | -289639 | 579615.1 | 579618 | 580948 |
| TPM3u+G(4)+I | 170 | -289639 | 579617.6 | 579620.6 | 580958.5 |
| TIM1e | 166 | -296805 | 593941.3 | 593944.2 | 595250.7 |
| TIM1e+I | 167 | -295491 | 591316.2 | 591319.1 | 592633.4 |
| TIM1e+G(4) | 167 | -289653 | 579639 | 579641.9 | 580956.2 |
| TIM1e+G(4)+I | 168 | -289653 | 579641.6 | 579644.5 | 580966.7 |
| TIM1 | 169 | -296800 | 593937 | 593939.9 | 595270 |
| TIM1+I | 170 | -295482 | 591303.6 | 591306.6 | 592644.5 |
| TIM1+G(4) | 170 | -289637 | 579613.7 | 579616.7 | 580954.5 |
| TIM1+G(4)+I | 171 | -289637 | 579616.2 | 579619.2 | 580965 |
| TIM2e | 166 | -296783 | 593897.6 | 593900.4 | 595206.9 |
| TIM2e+I | 167 | -295474 | 591282.5 | 591285.3 | 592599.7 |
| TIM2e+G(4) | 167 | -289637 | 579607.1 | 579610 | 580924.3 |
| TIM2e+G(4)+I | 168 | -289637 | 579609.7 | 579612.6 | 580934.7 |
| TIM2 | 169 | -296782 | 593901.4 | 593904.4 | 595234.4 |
| TIM2+I | 170 | -295472 | 591283.8 | 591286.8 | 592624.6 |
| TIM2+G(4) | 170 | -289631 | 579602.9 | 579605.9 | 580943.8 |
| TIM2+G(4)+I | 171 | -289632 | 579605.5 | 579608.5 | 580954.2 |
| TIM3e | 166 | -296804 | 593939.8 | 593942.6 | 595249.1 |
| TIM3e+I | 167 | -295492 | 591317.3 | 591320.1 | 592634.5 |
| TIM3e+G(4) | 167 | -289654 | 579641.3 | 579644.2 | 580958.5 |
| TIM3e+G(4)+I | 168 | -289654 | 579643.9 | 579646.8 | 580969 |
| TIM3 | 169 | -296797 | 593932.6 | 593935.5 | 595265.6 |
| TIM3+I | 170 | -295482 | 591304.2 | 591307.2 | 592645.1 |
| TIM3+G(4) | 170 | -289638 | 579616.6 | 579619.6 | 580957.5 |
| TIM3+G(4)+I | 171 | -289639 | 579619.2 | 579622.2 | 580967.9 |
| TVMe | 167 | -296783 | 593901 | 593903.8 | 595218.2 |
| TVMe+I | 168 | -295478 | 591291.4 | 591294.3 | 592616.5 |
| TVMe+G(4) | 168 | -289637 | 579610.3 | 579613.2 | 580935.4 |
| TVMe+G(4)+I | 169 | -289637 | 579612.9 | 579615.8 | 580945.9 |
| TVM | 170 | -296777 | 593894.8 | 593897.8 | 595235.7 |
| TVM+I | 171 | -295471 | 591283.9 | 591286.9 | 592632.7 |
| TVM+G(4) | 171 | -289630 | 579602.4 | 579605.4 | 580951.2 |
| TVM+G(4)+I | 172 | -289630 | 579605 | 579608 | 580961.6 |
| SYM | 168 | -296779 | 593893.2 | 593896.1 | 595218.3 |
| SYM+I | 169 | -295471 | 591280.7 | 591283.7 | 592613.7 |
| SYM+G(4) | 169 | -289635 | 579607.3 | 579610.2 | 580940.2 |
| SYM+G(4)+I | 170 | -289635 | 579609.8 | 579612.8 | 580950.7 |
| GTR | 171 | -296776 | 593894.1 | 593897.1 | 595242.9 |
| GTR+I | 172 | -295469 | 591281.6 | 591284.6 | 592638.2 |
| GTR+G(4) | 172 | -289630 | 579603.9 | 579606.9 | 580960.5 |
| GTR+G(4)+I | 173 | -289630 | 579606.4 | 579609.5 | 580971 |


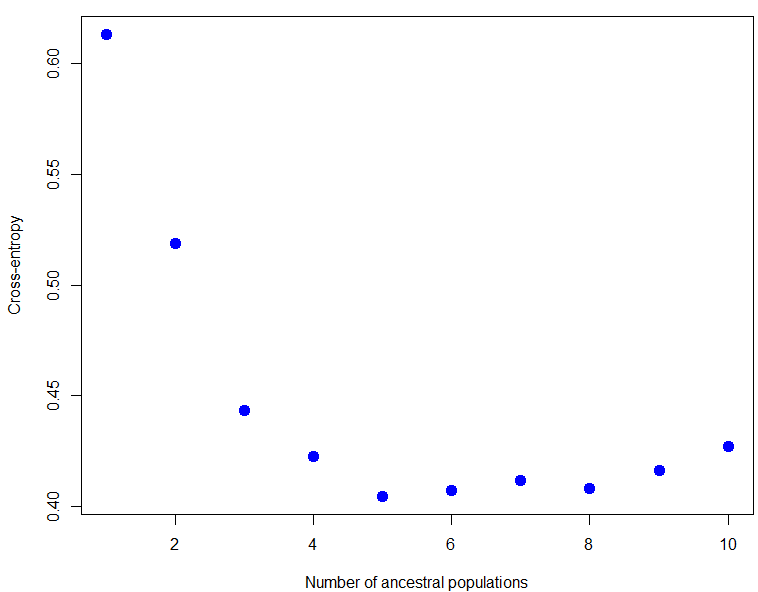


**Figure S1.** Cross-entropy plot to estimate individual assignments of three sandalwood species, *S. lanceolatum, S. leptocladum* and *S. macgregorii* to genetic groups (*K*) based on a sparse non-negative factorization method.

**Table S2.** Relative directional geneflow matrix (based on *G_ST_*) of three sandalwood species, *S. lanceolatum, S. leptocladum* and *S. macgregorii* analysed in regional groups. * indicates significant alpha values based on 0.5 threshold.

| **Regional groups** | *Santalum lanceolatum* | *Santalum lanceolatum NPA* | *Santalum leptocladum* | *Santalum macgregorii WP* | *Santalum macgregorii* |
| --- | --- | --- | --- | --- | --- |
| Santalum lanceolatum | 0.000 | 0.321 | 0.139 | 0.309 | 0.151 |
| Santalum lanceolatum NPA | 0.939* | 0.000 | 0.038 | 1* | 0.078 |
| Santalum leptocladum | 0.688* | 0.091* | 0.000 | 0.088 | 0.078 |
| Santalum macgregorii WP | 0.625* | 0.416 | 0.070 | 0.000 | 0.128 |
| Santalum macgregorii | 0.139 | 0.080 | 0.062 | 0.184* | 0.000 |
